# Weight lifting can facilitate appreciative comprehension for museum exhibits

**DOI:** 10.3389/fpsyg.2014.00307

**Published:** 2014-04-14

**Authors:** Yuki Yamada, Shinya Harada, Wonje Choi, Rika Fujino, Akinobu Tokunaga, YueYun Gao, Kayo Miura

**Affiliations:** ^1^Faculty of Arts and Science, Kyushu UniversityFukuoka, Japan; ^2^Graduate School of Human-Environment Studies, Kyushu UniversityFukuoka, Japan; ^3^Graduate School of Integrated Frontier Sciences, Kyushu UniversityFukuoka, Japan; ^4^Faculty of Human-Environment Studies, Kyushu UniversityFukuoka, Japan

**Keywords:** museology, memory, haptic, information integration, appreciation

## Abstract

Appreciation of exhibits in a museum can be equated to a virtual experience of lives in the contexts originally surrounding the exhibits. Here we focus on the importance of weight information, and hence tested whether experiencing a weight during museum exhibit appreciation affects the beholders' satisfaction and recognition memory for the exhibits. An experiment was performed at a museum exhibiting skeletal preparations of animals. We used nine preparations and prepared four weight stimuli as weight cues in accordance with the actual weight of four of the preparations: Remaining five preparations was displayed without weight stimuli. In the cued condition, participants were asked to lift up the weight stimuli during their observation of the four exhibits. In the uncued condition, participants observed the exhibits without touching the weight stimuli. After observation of the exhibits, the participants responded to a questionnaire that measured their impressions of the exhibits and the museum, and performed a recognition test on the exhibits. Results showed that memory performance was better and viewing duration was longer with weight lifting instruction than without instruction. A factor analysis on the questionnaires revealed four factors (likeability, contentment, value, and quality). A path analysis showed indirect effects of viewing duration on memory performance and willingness-to-pay (WTP) for the museum appreciation through the impression factors. Our findings provide insight into a new interactive exhibition that enables long appreciation producing positive effects on visitors' impression, memory, and value estimation for exhibits.

## Introduction

Through visiting museums, we are able to come into contact with things not usually seen in our daily lives, such as rare creatures, historical relics, archaeological remains, and artwork. Through the appreciations of museum exhibits, we can experience virtual lives in the spatial and temporal contexts that originally encompassed the exhibited items with vivid reality. Great progress in recent technologies related to virtual and augmented reality has allowed museums to incorporate three-dimensional (3D) representation into their exhibits (Walczak and White, 2003; Hirose, 2006; Petridis et al., 2006). Simultaneously, multimodal displays based on similar technology have been equipped to appeal to not only visual but also auditory, haptic, and olfactory modalities (Butler and Neave, 2008; Figueroa et al., 2009). Such new methods contribute to the diversity of presentation within and between museums, comprising a new category of “virtual museum” that distributes digital replicas of exhibits to each individual, going beyond the conventional static presentations that remain inside glass cases.

Multimodal displays are likely to facilitate a deeper understanding of museum exhibits. Indeed, it has been found that when haptic devices are incorporated into exhibits, visitors take more time to appreciate them (Butler and Neave, 2008); moreover, the inclusion of haptic devices in exhibits has received positive evaluations from not only visitors, but also museum curators and researchers (Asano et al., 2005). Thus, given that one of the objectives of museums is to educate visitors about their exhibits, it is important to improve visitors' impressions of exhibits and to encourage longer appreciation times by adding haptic information.

Directly touching museum exhibits can provide visitors with additional information on the texture, weight, and materials of items than merely viewing them can. In cognitive psychology, it has been suggested that the provision of more information about unknown things can elicit a positive affect (Biederman and Vessel, 2006; Yamada et al., 2012, 2013). These findings may underlie why the addition of haptic information to exhibits leads to more positive impressions of them. However, there is a dilemma in introducing such touchable museum exhibits. While exhibits have traditionally been susceptible to age-related natural deterioration and damage during transport, which can affect the condition of exhibit items to varying degrees, the touching of exhibit items by visitors' hands can also accelerate their deterioration.

To overcome this dilemma, in our study we focused on the addition of a simple object, a box, that has the same weight to exhibits. This is information for which “surrogates” (i.e., information that does not require the direct touching of original exhibit items) can be easily created and that does not seem to be directly related to positive or negative emotions regarding the exhibit. Moreover, to investigate such an addition experimentally, we can easily prepare and control the experimental stimuli when compared with texture and material stimuli. Humans perceive the weight of a stimulus based on outputs from proprioceptive and cutaneous sensors that constitute haptic perception (Jones, 1986; Flanagan et al., 1995; Flanagan and Wing, 1997). Moreover, many previous studies with psychophysics have revealed that haptic perception sometimes alters the appearance of visual stimuli (Ernst et al., 2000; Violentyev et al., 2005; Ide and Hidaka, 2013). Thus, we developed the hypothesis that weight cues will provide a portion of the available haptic information about museum exhibit items without direct touch, thereby resulting in positive effects on visitors' visual museum appreciation.

The goal of the present study was to examine whether weight cues affect visitor appreciation of museum exhibits. In our experiment, we prepared polystyrene foam boxes containing sand in accordance with the actual weight of each exhibit. We divided the participants into two conditions; in one, the participants had the opportunity to pick up the weight stimuli using their hands (cued condition), and in the other the participants had no opportunity to do so (uncued condition). We predicted that if our hypothesis was correct, that is, that weight cues affect participants' appreciation of exhibit items, then the participants in the cued condition would (1) have significantly more positive perceptions of the exhibit appreciation experience and of the museum itself, (2) be able to better recall a greater number of the exhibits, (3) look at exhibits for longer periods of time, and (4) be willing to pay more money to experience such appreciation when compared with the participants in the uncued condition.

## Materials and methods

### Participants

Forty-two graduate and undergraduate students attending Kyushu University participated in the experiment (12 men, 30 women; mean age = 21.7 years). The participants were unaware of the purpose of the experiment. The experiment was conducted according to the principles laid down in the Helsinki Declaration. Written informed consent was obtained from all participants after the nature and possible consequences of the study were explained to them. The ethical committees of Kyushu University approved the protocol. A total of 20 (3 men, 17 women) and 22 (9 men, 13 women) participants were randomly assigned to the cued and uncued conditions, respectively, which are described below.

### Stimuli

**Figure 1:**
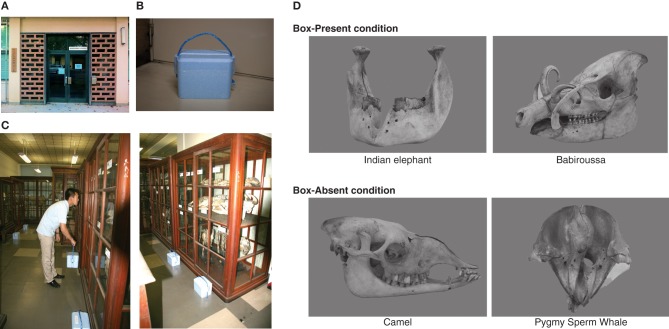
**(A)** Appearance of the first pavilion of The Kyushu University Museum. **(B)** A box of the weight cue used in the experiment. **(C)** Layout and use of the weight cues. **(D)** Examples of skeletal preparations used in the experiment.

The experiment was conducted at a museum belonging to the Kyushu University (Figure 1A; First pavilion of The Kyushu University Museum: http://www.museum.kyushu-u.ac.jp/english/index.html). An exhibition room displaying skeletal preparations of animals in glass cases was used (Figure 1C). There were nine skeletal preparations in total, and we presented weight stimuli with only four preparations (box-present condition), and hence, no weight stimuli were presented with the residual five preparations (box-absent condition). We used the following skeletal preparations, babirusa [*Babyrousa babyrussa*], Indian elephant [*Elephas maximus indicus*], short-finned pilot whale [*Globicephala macrorhyncus*], and water buffalo [*Bubalus arnee*], as exhibit items for the box-present condition and the following skeletal preparations, camel [*Camelidae*], reindeer [*Rangifer tarandus*], pygmy sperm whale [*Kogia breviceps*], sheep [*Ovis aries*], and sun bear [*Helarctos malayanus*], as exhibit items for the box-absent condition (Figure 1D). Participants both in the cued and uncued conditions appreciated all of these skeletal preparations. As a weight cue, we created weight stimuli with the same appearance but various mass values that corresponded to the actual weight of the four skeletal preparations (babirusa: 792.7 g; Indian elephant: 5114.6 g; short-finned pilot whale: 3276.4 g; water buffalo: 1554.7 g) used in the box-present condition (Figure 1B). The weight stimuli were blue polystyrene foam boxes that contained amounts of sand giving each box the appropriate respective weight. Each of these weight stimuli was placed in front of a skeletal presentation. For the five skeletal preparations in the box-absent condition, no box was prepared.

A paper-based questionnaire with a 7-point Likert-style scale (from 1 “I don't think so at all” to 7 “I strongly think so”) was employed. The questionnaire consisted of two parts. The first part included 14 questions regarding the participants' impressions of their museum appreciation experience, such as “*like*,” “*dislike*,” “*satisfied*,” “*dissatisfied*,” “*approachable*,” “*novel*,” “*surprised*,” “*boring*,” “*pleased*,” “*interesting*,” “*awful*,” “*exciting*,” “*enjoyable*,” and “*refreshing*.” The second part included 11 questions regarding the participants' impressions of the museum itself, such as “*scientific*,” “*better-than-expected*,” “*less-than-expected*,” “*dignified*,” “*ingenious*,” “*realistic*,” “*exhibits-enriched*,” “*recommendable to friends*,” “*increased my desire to learn*,” “*historic*,” and “*made me want to visit again*.”

### Procedure

Participants were instructed that they could freely view the exhibits in the museum. The participants assigned to the cued condition were additionally instructed as follows: “You'll find boxes in front of some exhibits. These boxes were prepared by the museum staff, and they have the same weight as the exhibit. Please make an effort to pick each box up to experience the actual weight of each exhibit.” On the other hand, the participants assigned to the uncued condition were instructed as follows: “You'll find boxes in front of some exhibits. These boxes contain documents available only to the museum staff. Please do not touch them.” After the visitors had experienced the museum exhibits, we asked them how much they were willing to pay, in Japanese yen, for the same museum experience (100 Japanese yen was nearly equivalent to one USD at the time of the experiment). Then, we carried out a memory (recognition) test on the names of the exhibits, with no time limit. The test items consisted of the nine items displayed in the museum, and 10 filler items that were not actually displayed (capybara, crocodile, giraffe, great Indian rhinoceros, hartebeest, hippopotamus, Malayan tapir, Reeves's muntjac, shearwater, and striped dolphin). Furthermore, we measured each participant's viewing duration with a stopwatch.

## Results

**Figure 2:**
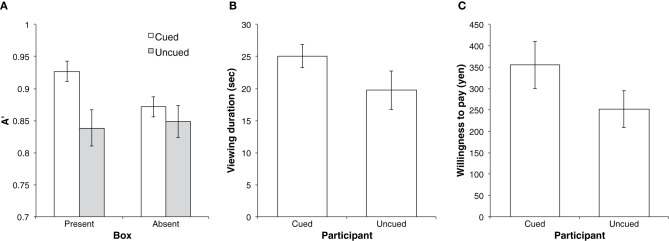
**The experimental results for (A) recognition performance, (B) viewing duration of each exhibit, and (C) willingness to pay**. Error bars denote standard errors of the means.

A post-experiment interview showed that one female participant had previously visited the museum, so data from her were not used for analysis. We compared the recognition performance, viewing duration, and willingness-to-pay (WTP) between the cued and uncued conditions. Recognition performance was indicated by A' (Figure 2A) and B”D measures (Donaldson, 1992). Viewing duration of each exhibit was calculated by dividing total duration by the number of exhibits (i.e., 19) and was analyzed after log-transformation (Figure 2B).

For the recognition performance, a mixed analysis of variance of the A' measure with cue condition as a between-subjects factor and box condition as a within-subjects factor was performed; results showed that only a main effect of cue was significant, *F*_(1, 39)_ = 5.31, *p* < 0.03, η^2^_*p*_ = 0.14. Although there was no interaction between cue and box conditions, we individually conducted paired *t*-tests and showed that the difference between the box-present and box-absent conditions was significant for the participants in the cued condition, *t*_(18)_ = 2.32, *p* < 0.04, Cohen's *d* = 0.82, while no significant difference between the conditions was found for the participants in the uncued condition, *t*_(21)_ = 0.29, *p* > 0.77, Cohen's *d* = 0.08. A two-sample *t*-test showed that the difference between the cued and uncued condition was significant for the box-present condition, *t*_(39)_ = 2.60, *p* < 0.02, Cohen's *d* = 0.85. On the other hand, ANOVA and individual *t*-tests did not show any significant effect in the B”D measure.

As for the viewing duration, a two-sample *t*-test revealed that participants in the cued condition viewed the exhibits significantly longer than participants in the uncued condition, *t*_(39)_ = 2.50, *p* < 0.02, Cohen's *d* = 0.80.

As for WTP, a two-sample *t*-test showed no significant difference between the cued and uncued conditions, *t*_(39)_ = 1.53, *p* > 0.13, Cohen's *d* = 0.49 (Figure 2C).

To investigate the relationship between the indices analyzed above and participants' impression of their exhibit experience as well as the museum, we used an exploratory factor analysis on the individual questionnaire data that extracted principal components with the unweighted least-square method and promax rotation after reversed items were adjusted. Bartlett's test of sphericity showed significant χ^2^ values [exhibit appreciation scale: χ^2^_(91)_ = 423.73, *p* < 0.0001; museum scale: χ^2^_(55)_ = 163.54, *p* < 0.0001]. The Kaiser-Meyer-Olkin measurement of sampling adequacy was equal to 0.78 and 0.67 for the exhibit appreciation and museum scales, respectively, suggesting that these data were suitable for factor analysis. Curves in the scree plot suggested that two factor solutions could be extracted from both the scales (Tables 1, 2). These factors explained 59.2% and 48.1% of the total variances in the exhibit appreciation and museum scales, respectively. For the participants' exhibit appreciation, the first and second factors included items on “likability” and “contentment” with their exhibit appreciation experience, respectively. For the impressions of the museum, the first and second factors included items on the “value” and “quality” of the museum, respectively.

**Table 1:** **Factor loadings for the items in the appreciation scale after promax rotation**.

**Item**		**Factor**		***h*^2^**
		**Likability**	**Contentment**	
Pleased		**0.948**	0.129	0.824
Interesting		**0.892**	0.116	0.777
Like		**0.868**	0.012	0.804
Enjoyable		**0.823**	−0.031	0.850
Dislike		**0.758**	−0.146	0.845
Refleshing		**0.750**	0.037	0.760
Approachable		**0.697**	0.066	0.569
Awful		**0.486**	−0.041	0.604
Dissatisfied		0.117	**0.769**	0.594
Satisfied		−0.325	**0.769**	0.806
Boring		0.030	**0.714**	0.555
Novel		−0.151	**−0.550**	0.481
Correlation	F1	1.000		
between factors	F2	−0.431	1.000	

*The bold values represent that these items constituted the corresponding factor*.

**Table 2:** **Factor loadings for the items in the museum scale after promax rotation**.

**Item**		**Factor**		***h*^2^**
		**Likability**	**Contentment**	
Made me want to visit again		**0.996**	−0.168	0.673
Recommendable to friends		**0.744**	0.240	0.747
Increased my desire to learn		**0.607**	0.094	0.514
Historic		**0.381**	0.058	0.282
Exhibits-enriched		−0.162	**0.908**	0.492
Better-than-expected		0.158	**0.625**	0.537
Dignified		0.113	**0.496**	0.479
Scientific		0.024	**0.478**	0.256
Less-than-expected		0.129	**0.450**	0.451
Correlation	F1	1.000		
between factors	F2	0.588	1.000	

*The bold values represent that these items constituted the corresponding factor*.

**Figure 3:**
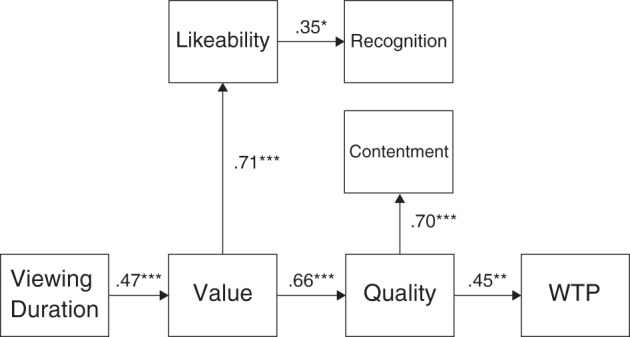
**Result of a path analysis**. The path coefficients represent standardized partial regression coefficient. ^*^p < 0.05; ^**^p < 0.01; ^***^p < 0.001.

Moreover, using each of the indices (recognition performance, viewing duration, and WTP) and the four factor scores, we performed a path analysis (Figure 3). The goodness of fit of a model to the data was high, χ^2^_(15)_ = 24.95, *p* > 05; RMR = 0.038; GFI = 0.856; AGFI = 0.731. We estimated 95% confidence intervals using a Bayesian framework with 100,000 iterations after a burn-in of 20,000 iterations. Through the resampling, all variables converged under the convergence criteria of 1.002 (Gelman et al., 2004). An indirect effect of the viewing duration on recognition performance and WTP was significant, 95% confidence intervals of the standardized indirect effects were 0.019 to 0.222 and 0.047 to 0.246, respectively.

Furthermore, we compared the four factor scores between the cued and uncued conditions. The results showed that there was no difference, suggesting that the manipulation of the weight cue did not directly affect the participants' impression.

Finally, since one can argue that any gender difference might have impacted on our results, we ran male vs. female comparisons with two-sample *t*-tests for viewing duration, recognition performance (A' and B”D values), and WTP. The results did not show any significant differences between males and females for viewing duration: *t*_(39)_ = 1.29, *p* > 0.20, Cohen's *d* = 0.45; A': *t*_(39)_ = 0.69, *p* > 0.49, Cohen's *d* = 0.24; B”D: *t*_(39)_ = 0.23, *p* > 0.81, Cohen's *d* = 0.08; nor WTP: *t*_(39)_ = 1.35, *p* > 0.18, Cohen's *d* = 0.47, and hence, the unexpected effect of gender on our results was foreclosed.

## Discussion

The results of the mean differences between the groups based on the presence of weight cueing suggest significant effects of cue for each behavioral index. First, participants who were instructed to lift the weights showed significantly higher memory performance relative to the other participants. Although this was not confirmed under the significant interaction, the cued participant's memory performance of the box-present exhibits was higher than that of the box-absent exhibits. This effect was not due to a gender difference. Moreover, considering all the participants viewed the boxes, the explanation that the box merely served as a visual marker for memory retrieval does not seem plausible. Instead, the results might come from the instruction itself in a manner of increasing the participants' arousal or motivation to the appreciation.

Second, consistent with the results of a previous study (Butler and Neave, 2008), our results showed that participants with access to haptic experience took a significantly longer time to appreciate exhibits than participants without such haptic experience access. One could argue that this time difference reflects the time spent on the lifting action itself. The mean difference of the total viewing duration between groups was approximately 106.6 s and the total duration required for lifting action that was measured in a supplementary experiment was approximately 19.7 s (see Appendix). Even when this lifting duration was subtracted from the total viewing duration in the cued condition, the significant difference between the cued and uncued condition in viewing duration per exhibit was preserved, *t*_(39)_ = 2.26, *p* < 0.04, Cohen's *d* = 0.71. Therefore, it is unlikely that merely the lifting action of four boxes produced the difference observed in the main experiment. Third, the difference in the WTP variable 102.99 Japanese yen higher in the cued condition did not reach significance, suggesting that the weight cue alone was not sufficient to significantly change the participants' WTP directly.

Analyses of the questionnaire data provided much evidence supporting the group differences. The factor analysis showed that the questionnaire items could be categorized into two principal factors: the factors “likability” and “contentment” with respect to the participants' impressions of the exhibit appreciation experience, and factors “value” and “quality” with respect to the participants' impressions of the museum itself. The results of the path analysis suggest that the formation of participants' impressions regarding viewing the museum exhibits was influenced by likability-based processing regarding exhibit appreciation and quality-based processing regarding the museum itself after establishing the impression of value. One implication of these results is that curators' efforts to facilitate the visitors' memories of exhibits may not necessarily lead to their positive attitudes toward paying more for similar museum experiences. Our findings of no significant correlation between WTP and recognition performance, *r* = 0.02, *p* > 0.91, support this implication.

How did the weight cues affect the results? In light of the internal mechanisms of participants, as mentioned in the introduction, we first surmised that the greater amount of information provided from multiple modalities may lead to higher processing fluency for the corresponding objects, thereby enhancing the esthetic pleasure experienced through perceiving those objects (Reber et al., 1998, 2004; Kuchinke et al., 2009). However, this might not be true or at lease it is somewhat premature to draw a conclusion on this issue, because the results showed that the manipulation of the weight cue did not directly affect any factor scores. The results of the path analysis instead indicate another possibility. We just found that the weight cue significantly prolonged the viewing duration and a model with the viewing duration as a causal factor could explain the results. Hence, an indirect effect of the manipulation of the weight cue might exist. That is, the lifting action prolongs the viewing duration, and then the viewing duration changes participants' impression of exhibits and museum (for example, a significant direct effect of viewing duration on value was found), and then these impression changes affect recognition performance and WTP through separate paths. These findings have important implication on a future museum exhibition: Interaction with even a box with a neutral appearance does change visitors' impression, memory, and value estimation for exhibits.

In conclusion, both the data for each behavioral index and the questionnaire data indicate that the interaction with physical surrogate objects is (at least indirectly) sufficient to facilitate visitors' appreciation of museum exhibits. Future studies should address to what degree the weight information is important. For example, one could hypothesize that weight information does not contribute to the appreciation of pictorial art exhibit items because the esthetic value of such paintings is obviously unrelated to their physical weight. Furthermore, it is unclear whether weight information itself is important. Although visitors do not know the actual weight of the exhibits, visitors can also guess somewhat the weight of the exhibits on the basis of the visually perceived size and texture of them. Hence, a loose matching between the weight cues and the exhibits might matter. Moreover, depending on the shape of the exhibits, there is a difference in the weight distribution between the weight cues and the exhibits. How these issues influence the visitors' appreciation should be clarified in future. Owing to the recent progress in 3D printing technology, 3D printers can now easily produce precise replicas of all kinds of items for application in museum exhibits (Allard et al., 2005; Kelley et al., 2007; Niven et al., 2009). Such surrogate exhibits allow visitors to directly touch items freely without having to worry about damaging the original items. The 3D replica-based experiments would resolve the issues about weight information we discussed above. Further cross-disciplinary investigations between museology and psychology that focus on the role of such surrogate exhibit items are needed for a deeper understanding of not only the mental mechanisms involved in the appreciation of museum exhibits, but also their creation and presentation.

### Conflict of interest statement

The authors declare that the research was conducted in the absence of any commercial or financial relationships that could be construed as a potential conflict of interest.

## Appendix

To support our dismissal of the possible contamination of lifting-time itself, we additionally conducted an experiment that measured the time needed to the lifting action. In this supplementary experiment, participants were asked to freely lift up the boxes used in the main experiment under the instruction “Please freely lift all the boxes up.” This experiment was conducted in an indoor space of Kyushu University. The stimuli were same as the weight cues (boxes) used in the main experiment. Other than the weight cues, there was neither object nor exhibit. We measured the lifting time via stopwatch. Eighteen participants took part in this experiment (5 men, 13 women; mean age = 22.3 years). The results showed that the total lifting duration was 19.66 s (*SD* = 3.62 s). This lifting duration was subtracted from the total viewing duration in the cued condition. Even so, there was the significant difference between the cued and uncued condition in the viewing duration per exhibit, *t*_(39)_ = 2.26, *p* < 0.04, Cohen's *d* = 0.71. This, we dismissed the possibility that merely the lifting action of four boxes produced the difference in the main experiment.

## References

[B1] Allard T. T. Sitchon M. Sawatzky R. Hoppa R. D. (2005). Use of hand-held laser scanning and 3D printing for creation of a museum exhibit, in Proceedings of the 6th International Symposium on Virtual Reality, Archaeology and Cultural Heritage: Short and Project Papers, eds Mudge M. Ryan N. Scopigno R. (Pisa), 97–101

[B2] Asano T. Ishibashi Y. Minezawa S. Fujimoto M. (2005). Surveys of exhibition planners and visitors about a distributed haptic museum, in Proceedings of the 2005 ACM SIGCHI International Conference on Advances in Computer Entertainment Technology (New York, NY: ACM), 246–249 10.1145/1178477.1178518

[B3] Biederman I. Vessel E. (2006). Perceptual pleasure and the brain: a novel theory explains why the brain craves information and seeks it through the senses. Am. Sci. 94, 247–253 10.1511/2006.59.995

[B4] Butler M. Neave P. (2008). Object appreciation through haptic interaction, in Hello! Where are you in the landscape of educational technology? Proceedings Ascilite Melbourne 2008 (Melbourne, VIC), 133–141

[B5] Donaldson W. (1992). Measuring recognition memory. J. Exp. Psychol. Gen. 121, 275–277 10.1037/0096-3445.121.3.275 1402701

[B6] Ernst M. O. Banks M. S. Bülthoff H. H. (2000). Touch can change visual slant perception. Nat. Neurosci. 3, 69–73 10.1038/71140 10607397

[B7] Figueroa P. Coral M. Boulanger P. Borda J. Londoño E. Vega F. (2009). Multi-modal exploration of small artifacts: an exhibition at the Gold Museum in Bogota, in Proceedings of the 16th ACM Symposium on Virtual Reality Software and Technology (Kyoto), 67–74

[B8] Flanagan J. R. Wing A. M. (1997). Effects of surface texture and grip force on the discrimination of hand-held loads. Percept. Psychophys. 59, 111–118 10.3758/BF03206853 9038413

[B9] Flanagan J. R. Wing A. M. Allison S. Spenceley A. (1995). Effects of surface texture on weight perception when lifting objects with a precision grip. Percept. Psychophys. 57, 282–290 10.3758/BF03213054 7770320

[B10] Gelman A. Carlin J. Stern H. Rubin D. (2004). Bayesian Data Analysis. New York, NY: Chapman & Hall

[B11] Hirose M. (2006). Virtual reality technology and museum exhibit. Int. J. Virtual Real. 5, 31–36 10.1007/11590361_1

[B12] Ide M. Hidaka S. (2013). Tactile stimulation can suppress visual perception. Sci. Rep. 3:3453 10.1038/srep03453 24336391 PMC3861798

[B13] Jones L. A. (1986). Perception of force and weight: theory and research. Psychol. Bull. 100, 29–42 10.1037/0033-2909.100.1.29 2942958

[B14] Kelley D. J. Farhoud M. Meyerand M. E. Nelson D. L. Ramirez L. F. Dempsey R. J. (2007). Creating physical 3D stereolithograph models of brain and skull. PLoS ONE 2:e1119 10.1371/journal.pone.0001119.s002 17971879 PMC2040197

[B15] Kuchinke L. Trapp S. Jacobs A. M. Leder H. (2009). Pupillary responses in art appreciation: effects of aesthetic emotions. Psychol. Aesthet. Creat. Arts 3, 156–163 10.1037/a0014464

[B16] Niven L. Steele T. E. Finke H. Gernat T. Hublin J.-J. (2009). Virtual skeletons: using a structured light scanner to create a 3D faunal comparative collection. J. Archaeol. Sci. 36, 2018–2023 10.1016/j.jas.2009.05.021

[B17] Petridis P. Mania K. Pletinckx D. White M. (2006). The EPOCH multimodal interface for interacting with digital heritage artefacts. Lect. Notes Comput. Sci. 4270, 408–417 10.1007/11890881_45

[B18] Reber R. Schwarz N. Winkielman P. (2004). Processing fluency and aesthetic pleasure: is beauty in the perceiver's processing experience? Pers. Soc. Psychol. Rev. 8, 364–382 10.1207/s15327957pspr0804_3 15582859

[B19] Reber R. Winkielman P. Schwarz N. (1998). Effects of perceptual fluency on affective judgments. Psychol. Sci. 9, 45–48 10.1111/1467-9280.00008

[B20] Violentyev A. Shimojo S. Shams L. (2005). Touch-induced visual illusion. Neuroreport 16, 1107–1110 10.1097/00001756-200507130-00015 15973157

[B21] Walczak K. White M. (2003). Cultural heritage applications of virtual reality, in Web3D '03: Proceedings of the Eighth International Conference on 3D Web Technology (New York, NY: ACM), 182–183 10.1145/636593.636623

[B22] Yamada Y. Kawabe T. Ihaya K. (2012). Can you eat it? A link between categorization difficulty and food likability. Adv. Cogn. Psychol. 8, 248–254 10.2478/v10053-008-0120-2 22956990 PMC3434679

[B23] Yamada Y. Kawabe T. Ihaya K. (2013). Categorization difficulty is associated with negative evaluation in the “uncanny valley” phenomenon. Jpn. Psychol. Res. 55, 20–32 10.1111/j.1468-5884.2012.00538.x

